# *In vivo* immune engineering via mRNA therapeutics: reprogramming the post-infarction cardiac microenvironment

**DOI:** 10.3389/fimmu.2026.1873905

**Published:** 2026-06-09

**Authors:** Yiying Liu, Ruikang Liu, Chao Meng, Jun Li, Kai Yang, Fuyuan Zhang, Xiao Xia, Guancheng Ye, Yulian Yuan

**Affiliations:** 1 Department of Cardiology, Guang’anmen Hospital, China Academy of Chinese Medical Sciences, Beijing, China; 2 Graduate School, Beijing University of Chinese Medicine, Beijing, China; 3 Department of Rheumatology, Dongzhimen Hospital, Beijing University of Chinese Medicine, Beijing, China; 4 School of Medicine, Tsinghua University, Beijing, China; 5 Yuquan Hospital, Tsinghua University, Beijing, China

**Keywords:** myocardial infarction, mRNA therapeutics, lipid nanoparticles, immune reprogramming, macrophage polarization, immunometabolism, trained immunity, cardiac fibrosis

## Abstract

Myocardial infarction (MI) initiates a biphasic immune response, which plays a critical role in determining whether the heart undergoes adaptive repair or progresses to pathological fibrosis. Traditional drug and gene therapy delivery systems have been insufficient in precisely modulating this intricate sequence of events in both temporal and spatial dimensions. In recent years, nucleoside-modified messenger RNA (mRNA) technology, encapsulated within lipid nanoparticles (LNPs), has emerged as a novel platform for delivering transient, non-integrating, and repeatable immune modulation to the injured heart. This article will explore recent interdisciplinary advancements in mRNA technology and cardiac immunology from five distinct perspectives. (i) Strategies for mRNA design, encompassing nucleoside modifications and purification techniques, are primarily aimed at circumventing detection by innate immune sensors within inflamed myocardial tissue; (ii) The concept of trained immunity is investigated, focusing on how transient expression of mRNA encoding epigenetic editors may potentially erase pathological epigenetic imprints in myeloid progenitor cells; (iii) Immune cell reprogramming is examined, addressing the myeloid lineage through macrophage polarization and the degradation of neutrophil extracellular traps via metabolic and transcriptional reprogramming, as well as the lymphoid lineage through transient CAR-T cells, in situ-induced regulatory T cells, and regulatory B cells, with an emphasis on the role of cardiomyocytes as paracrine signaling hubs; (iv) The optimization of lipid nanoparticle (LNP) delivery technology is discussed, including SORT-based organ-targeting strategies and the immunogenicity challenges faced by lipid carriers in ischemic tissues, alongside the development of next-generation self-amplifying RNA payloads; (v) Standards for clinical translation are outlined, involving representative pipelines such as AZD8601 and mRNA-0184, strategies for repeated dosing from an immunological perspective, and the use of biomarkers to guide precision dosing timing. In conclusion, we propose an innovative framework that integrates spatial transcriptomics, gender-stratified dosing strategies, and multi-mRNA formulation technology, with the objective of establishing a viable pathway for personalized cardiac immune reprogramming.

## Graphical Abstract

**Figure:**
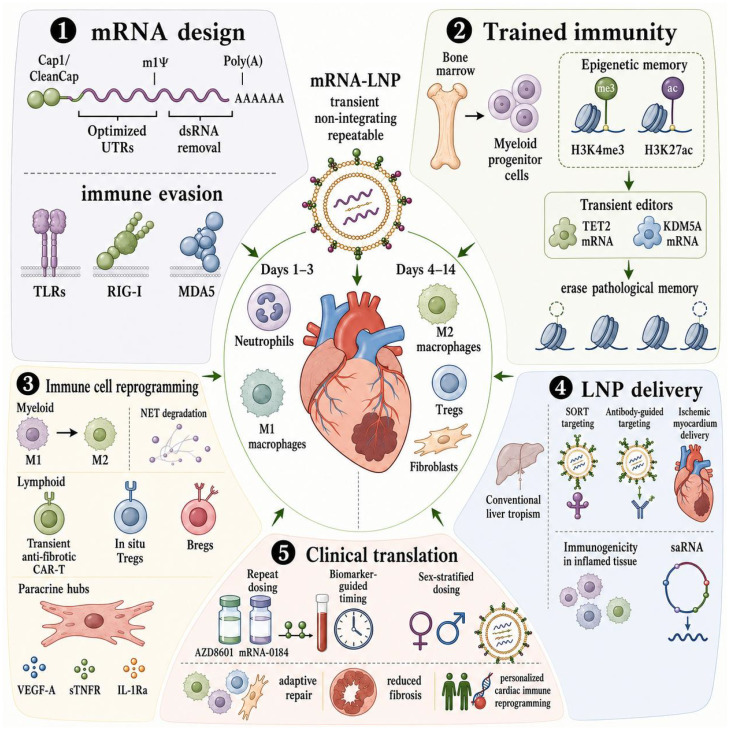

## 1 Introduction

Acute myocardial infarction (MI) continues to be a predominant cause of morbidity and mortality on a global scale, with ischemic heart disease responsible for approximately 9 million deaths annually (1). Beyond the immediate damage inflicted by acute ischemia, the ensuing inflammatory cascade plays a pivotal role in determining whether the ventricular tissue will heal effectively or progressively undergo remodeling, potentially leading to heart failure (2). Importantly, the immune response following an infarction is dynamic, characterized by a precisely orchestrated biphasic pattern: during the initial pro-inflammatory phase (days 1–3), there is a substantial influx of neutrophils into the infarcted zone, and classically activated (M1) macrophages predominate in the clearance of necrotic tissue. This phase is succeeded by the repair phase (days 4–14), wherein macrophages gradually shift towards an anti-inflammatory (M2) phenotype, regulatory T cells (Tregs) proliferate, and fibroblasts commence the formation of scar tissue (3, 4). The regulation of this temporal window is critical; premature suppression of early inflammation or delayed resolution of the inflammatory response can result in severe clinical outcomes, such as ventricular rupture or expansion of the infarcted area (5).

Traditional therapeutic approaches frequently prove inadequate in managing the intricate balance of the immune system. For instance, small-molecule anti-inflammatory drugs such as colchicine and methotrexate exhibit broad immunosuppressive effects but lack temporal specificity. The CANTOS trial demonstrated that canakinumab’s neutralization of interleukin-1β (IL-1β) effectively reduces the incidence of cardiovascular events; however, it also highlighted an increased vulnerability to severe infections, emphasizing the inherent risks of prolonged immunosuppression (6, 7). In the context of gene therapy, the decision between transient and sustained gene expression is a critical therapeutic consideration. Adeno-associated virus (AAV) vectors facilitate robust, long-term transgene expression, rendering them highly suitable for the treatment of monogenic cardiomyopathies that require lifelong correction (8). Nevertheless, this permanence poses significant challenges in the dynamic environment following MI. The prolonged or permanent expression of anti-inflammatory genes through AAV vectors can interfere with the critical transition from the inflammatory phase to the reparative phase, potentially hindering normal scar formation and increasing the risk of ventricular rupture (9). Similarly, although CRISPR-Cas9 genome editing provides an unprecedented level of precision and permanence in the ablation of stable pathogenic targets, such as PCSK9 mutations or oxidized CaMKIIδ, it lacks the temporal flexibility necessary to orchestrate the highly reversible and phase-specific biphasic immune responses following MI (10, 11). Moreover, AAV-mediated therapies encounter significant practical challenges: more than 50% of the human population has pre-existing neutralizing antibodies against AAV capsids, high-dose systemic delivery poses a risk of severe hepatotoxicity, and the permanent adaptive immune response against the viral capsid fundamentally precludes repeat dosing (9, 12). Consequently, the post-MI context necessitates an immunomodulatory intervention that is highly transient, titratable, and capable of being redosed, rather than relying on permanent genetic modification.

The advent of nucleoside-modified mRNA therapies presents a promising approach to addressing the previously mentioned limitations (13, 14). In particular, synthetic mRNA encoding therapeutic proteins can be encapsulated within lipid nanoparticles (LNPs), facilitating transient protein expression 24 to 72 hours post-administration. As mRNA inherently lacks the capacity to integrate into the genome, it circumvents the immunogenicity issues typically associated with viral vectors, thereby permitting repeated dosing (15). Crucially, nucleoside modifications, especially the incorporation of N1-methylpseudouridine (m1Ψ), effectively conceal the mRNA from detection by the body’s early innate immune sensors. This pivotal discovery has enabled mRNA therapy to extend beyond the singular application of vaccination, establishing it as a viable option across a broader spectrum of therapeutic domains (16, 17). The successful clinical validation of COVID-19 mRNA vaccines (BNT162b2 and mRNA-1273) has rapidly accelerated translational research into their application for various diseases, with cardiovascular disease treatment emerging as a significant area of focus (18).

**Figure 1:**
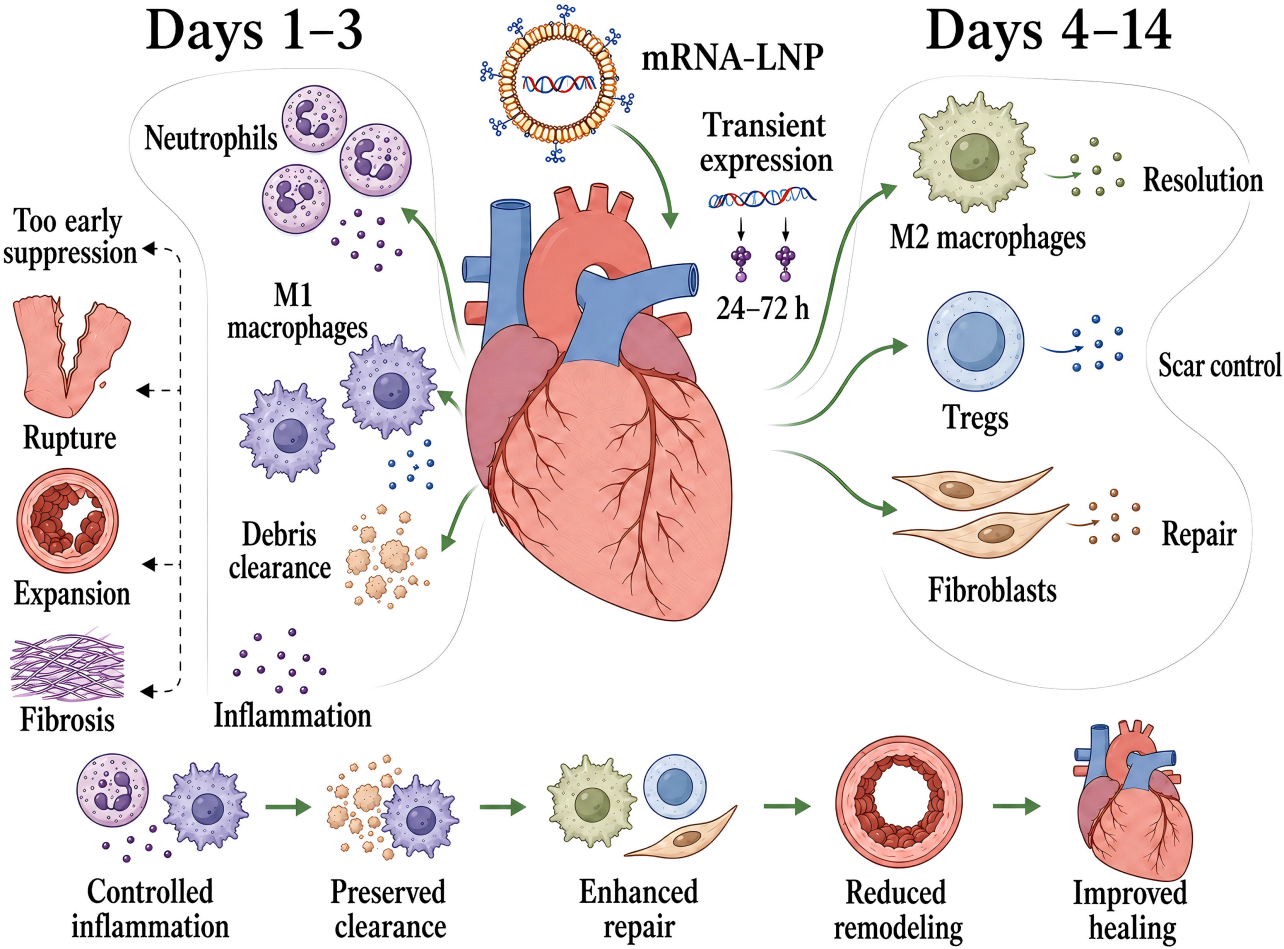
The biphasic immune response following myocardial infarction and windows for mRNA-LNP mediated intervention. The biphasic nature of the post-MI immune response requires precise timing. The transient expression kinetics of mRNA-LNPs (24–72 h) allow for phase-matched interventions, preventing adverse outcomes associated with premature or prolonged immunosuppression.

This review systematically examines mRNA-LNP therapy from an immunological standpoint, emphasizing its mechanisms of action in cardiac repair post-myocardial infarction. We introduce a conceptual framework termed ‘spatiotemporal immune reprogramming’, which aims to harness the transient expression properties of mRNA to align precisely with the immune regulatory demands at various stages of myocardial repair (**Figure 1**). The article further investigates specific technical methodologies for *in vivo* immune cell engineering, including the development of transient anti-fibrotic CAR-T cells and the targeted modulation of *in situ* regulatory Tregs. Notably, when LNPs are administered to ischemic myocardial tissue, where an inflammatory response is already present, an often-overlooked immunogenicity paradox arises, which we have also analyzed. Through this comprehensive and multi-dimensional discussion, we seek to delineate a clear trajectory for the advancement of mRNA-based cardiac immunotherapy from preclinical research to clinical application (**Table 1**).

**Table 1:** Comparison of therapeutic platforms for cardiac immunomodulation.

Feature	Small molecules (6, 7)	AAV gene therapy (8, 19)	mRNA-LNP (13, 14)	saRNA-LNP (20, 21)
Expression Duration	Hours (systemic)	Months–years	24–72 hours	1–2 weeks
Genomic Integration	N/A	Rare but possible	None	None
Repeat Dosing	Daily required	Limited (anti-capsid Ab)	Feasible	Feasible (with IFN mitigation)
Temporal Precision	Low	Very low	High	Moderate
Cell-Specific Targeting	Low	Moderate (serotype)	High (Ab-conjugated LNP)	High
*In Vivo* Cell Reprogramming	Not possible	Possible but permanent	Possible and transient	Possible, extended window
Immunogenicity	Low	High (capsid)	Moderate (LNP)	Higher (dsRNA intermediates)

## 2 Overcoming innate immune barrier: mRNA design for the inflamed heart

The development of mRNA therapies for post-myocardial infarction cardiac tissue must initially address a significant immunological challenge: the innate immune milieu of ischemic myocardium is notably hostile. Dying cardiomyocytes release damage-associated molecular patterns (DAMPs), such as HMGB1, mitochondrial DNA, ATP, and S100 proteins. These molecules initiate sterile inflammation by activating pattern recognition receptors (PRRs) on both resident and recruited immune cells, thereby resulting in heightened innate immune surveillance (22, 23). The introduction of exogenous RNA into this already highly sensitive inflammatory environment is likely to further exacerbate the inflammatory cascade. Consequently, optimizing mRNA design transcends mere pharmacological fine-tuning; it is a crucial determinant of treatment safety.

### 2.1 Nucleoside modifications and immune evasion

Pioneering research by Karikó and Weissman demonstrated that incorporating naturally occurring modified nucleotides, particularly pseudouridine (Ψ) and m1Ψ, into synthetic mRNA significantly diminishes the recognition efficiency of endosomal Toll-like receptors (TLR3, TLR7, TLR8) and cytoplasmic RNA sensors (RIG-I, MDA5) (16, 24).

These modifications effectively inhibit the activation of IFN-I response, which is particularly crucial in the inflammatory milieu following myocardial infarction. This is due to the fact that IFN-α and IFN-β frequently exert widespread and predominantly deleterious effects in this context: the IFN-I signaling pathway perpetuates the activation of M1 macrophages, hinders the differentiation of fibroblasts into a reparative phenotype, and obstructs neovascularization, all of which are detrimental to the cardiac healing process (25, 26).

Currently, m1Ψ has emerged as the preferred modification for therapeutic mRNA, as it surpasses pseudouridine in both translation efficiency and immunogenicity control (27). Research conducted by Andries et al. demonstrated that in mammalian cells, the expression level of proteins encoded by m1Ψ-modified mRNA can be approximately 13 times higher than that of unmodified mRNA, while the induction of cytokines mediated by TLRs is nearly completely suppressed (27). The enhancement in translation efficiency is largely attributed to improved ribosome loading kinetics and the reduced activity of protein kinase R (PKR) and 2′-5′-oligoadenylate synthetase (OAS), both of which are interferon-induced and function to inhibit translation (28). This modification holds particular significance in the field of cardiac research, as PKR activation in cardiomyocytes also initiates apoptotic signaling through eIF2α phosphorylation (29).

### 2.2 mRNA purification and structural optimization

Beyond nucleoside modification, the removal of dsRNA contaminants generated during *in vitro* transcription (IVT) is equally crucial. dsRNA serves as a potent activator of RIG-I and MDA5; if not removed, it can negate the immunological advantages conferred by nucleoside modification (30). The elimination of these byproducts through high-performance liquid chromatography (HPLC) or cellulose-based purification techniques can enhance protein expression in dendritic cells by nearly 1,000-fold, while simultaneously reducing the induction of IFN-I to nearly undetectable levels (24). This purification step is particularly crucial in cardiac research, given that myocardial tissue is inherently in an inflammatory state, and dsRNA may further activate the NLRP3 inflammasome, a pathway that is already significantly upregulated following myocardial infarction (31).

Additionally, the structural optimization of mRNA transcripts can modulate their expression kinetics and immunogenicity. The 5′ cap structure (Cap1 or CleanCap) influences not only the efficiency of translation initiation but also the resistance to cap-removing enzymes (32). The combined optimization of the 5′ and 3′ untranslated regions (UTRs), codon selection within the coding sequence, and the length of the polyadenylation tail collectively determine mRNA stability, translation efficiency, and the duration of protein expression (33). In cardiac applications, it is crucial to highlight that the length of the polyadenylated tail functions as a regulatory mechanism for fine-tuning gene expression; specifically, a shorter tail sequence facilitates more transient expression, which may be beneficial during the pro-inflammatory phase of interventions. This is because extended immune modulation during this phase could result in detrimental outcomes (34).

### 2.3 IFN response and its consequences for cardiac repair

Despite advancements in optimizing mRNA design, it remains imperative to exercise caution concerning residual effects of innate immune activation within the cardiac milieu. Research conducted by King et al. demonstrates that the IFN-I signaling pathway, activated post-myocardial infarction, enhances the recruitment of CCR2^+^ monocytes to the infarct area while simultaneously inhibiting the phenotypic transition of reparative macrophages, thereby aggravating ventricular remodeling (26). Consequently, any mRNA-based therapy that inadvertently induces IFN-I production may paradoxically lead to a worsened prognosis. This suggests that in preclinical models of MI, a comprehensive evaluation of the cytokine profile elicited by mRNA is essential. However, many contemporary pharmacological studies continue to depend on conventional assessment methods used for untreated animals, a practice that necessitates refinement.

Additionally, there may be a potential positive feedback loop between mRNA-induced innate immune activation and pre-existing DAMP-driven inflammation. Mitochondrial DNA released from damaged cardiomyocytes activates the cGAS-STING pathway, thereby placing the innate immune system in a pre-activated state, which likely reduces the threshold for mRNA-IFN-I responses (35, 36). This pre-activation effect indicates that mRNA design must consider the sensitivity to inflammation. When optimizing therapies, it is imperative to fully consider the pre-existing level of innate immune activation in the target tissue, rather than evaluating the immunogenicity of mRNA in isolation.

### 2.4 Trained immunity: can mRNA erase pathological epigenetic memory?

Beyond acute innate immune responses, the long-term cardiac prognosis following myocardial infarction is also affected by a more subtle yet equally detrimental process known as trained immunity. The concept initially proposed by Netea et al. involves a core mechanism centered on the epigenetic reprogramming of monocytes and macrophages through histone modifications, specifically H3K4 trimethylation (H3K4me3) and H3K27 acetylation (H3K27ac) at the promoter regions of pro-inflammatory genes, including TNF-α, IL-6, and IL-1β (37). In contrast to the classical lymphocyte-mediated immune memory, trained immunity operates at the level of myeloid progenitor cells within the bone marrow and can persist for several weeks to months following the initial insult (38).

There is an increasing body of evidence supporting the occurrence of immune training post-myocardial infarction. Research has demonstrated that monocytes isolated from patients several months after experiencing a myocardial infarction exhibit an exaggerated inflammatory response upon re-stimulation, characterized by significantly elevated production of TNF-α and IL-6 compared to healthy controls (39). This phenomenon of epigenetic scarring originates from hematopoietic stem and progenitor cells (HSPCs) in the bone marrow, which undergo reprogramming in response to stimuli such as β-glucan or oxidized LDL, thereby continuously supplying preactivated monocytes to the circulation (40). Recent mechanistic studies have significantly advanced our understanding of this process. For example, Dong has demonstrated that MI induces a trained immunity phenotype in bone marrow-derived monocyte macrophages through selective epigenetic reprogramming, which systematically accelerates subsequent atherogenesis and systemic inflammation (41). Clinically, this results in a self-perpetuating cycle: these ‘trained’ monocytes, once recruited to the healing myocardium, sustain a state of low-grade inflammation, thereby exacerbating fibrosis and hastening the progression of heart failure.

mRNA therapy presents a promising avenue for disrupting this cycle through multiple mechanisms. One strategy employs SORT lipid nanoparticles designed to target the spleen, facilitating the delivery of mRNA encoding TET2 (ten-eleven translocation methylcytosine dioxygenase 2) to monocytes in the bone marrow. TET2 plays a critical role by catalyzing the demethylation of DNA at loci associated with inflammatory genes, and its functional loss during clonal hematopoiesis has been implicated in triggering severe inflammatory responses following myocardial infarction (42). By temporarily restoring TET2 activity via mRNA, it is possible to ‘reset’ the epigenetic state of trained progenitor cells without inducing permanent genetic alterations. A second strategy focuses on histone demethylases: mRNA encoding KDM5A, which removes the active H3K4me3 mark, can mitigate this trained phenotype at the chromatin level. The transient nature of mRNA expression is particularly beneficial in this context; through repeated low-dose administrations over several consecutive weeks, pathological epigenetic memory can be incrementally erased, a feat that is challenging to achieve with constitutive gene therapy (43).

## 3 mRNA-mediated immune cell reprogramming in the post-MI heart

**Figure 2:**
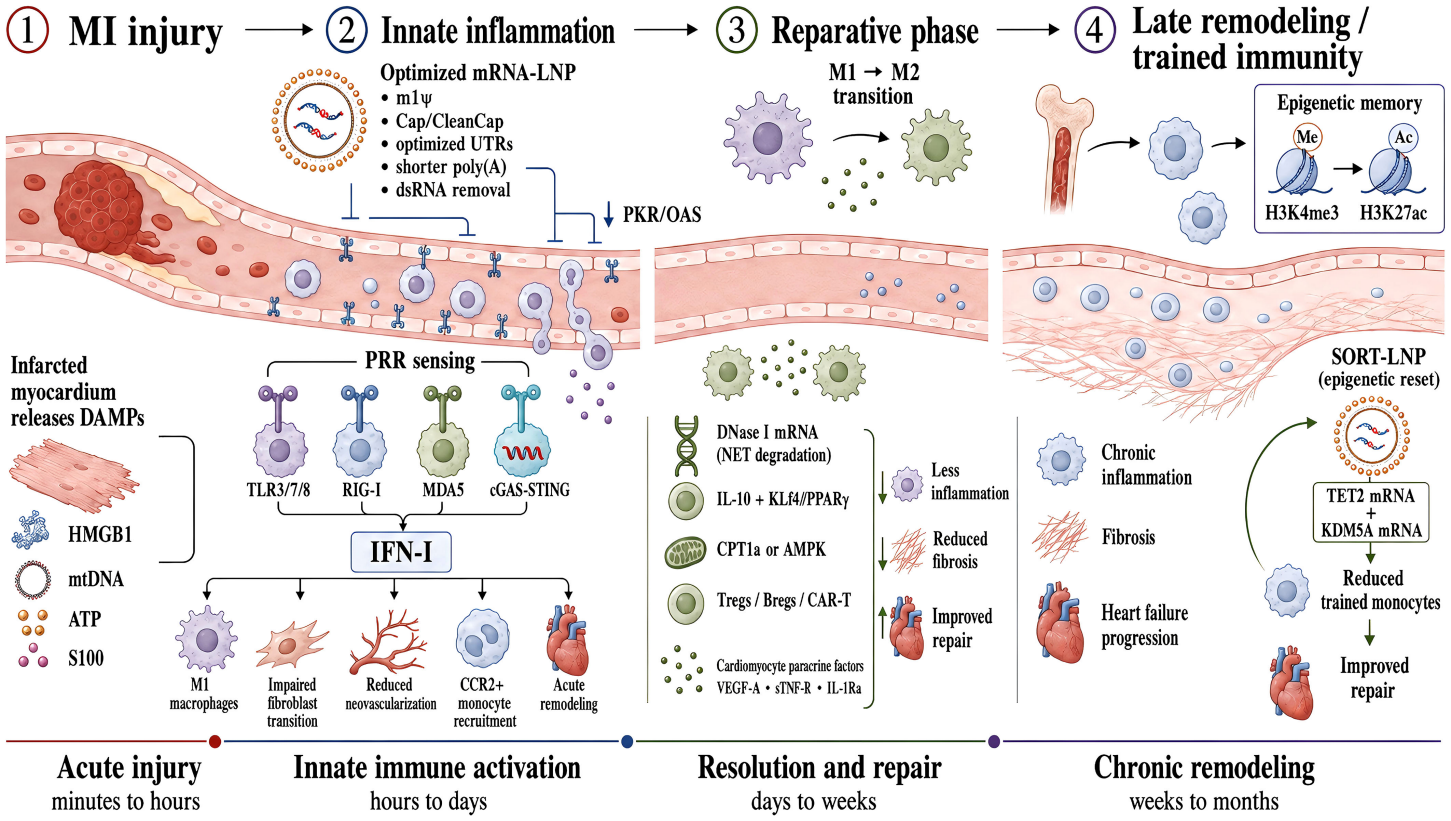
Molecular mechanisms and targets of mRNA-LNP therapies across different phases of post-MI cardiac repair. Optimized mRNA designs mitigate detrimental IFN-I signaling during early innate immune sensing (Phases 1-2). Specific mRNA payloads then drive immune cell reprogramming to enhance the M1-to-M2 transition and tissue repair (Phase 3), while epigenetic editing (e.g., TET2 mRNA) addresses chronic maladaptive trained immunity in the bone marrow (Phase 4).

The *in vivo* reprogramming of endogenous immune cells utilizing mRNA-LNP represents one of the most promising applications of this technology within the cardiovascular domain. Unlike conventional methods that merely facilitate the expression of therapeutic proteins, this innovative approach harnesses the translational machinery of the patient’s own immune cells to generate engineered effector cells or regulatory cell populations characterized by well-defined specificity and temporally regulated activity (**Figure 2**). The subsequent discussion will categorize this approach based on target cell types, specifically myeloid cells, lymphocytes, and cardiomyocytes, which function as paracrine hubs.

### 3.1 Myeloid targets: macrophage polarization, NET modulation, and immunometabolism

In the context of the post-myocardial infarction heart, myeloid cells, including monocytes, macrophages, and neutrophils, constitute a predominant component of the innate immune system, rendering them highly accessible targets for mRNA-based interventions (44, 45). Cardiac macrophages exhibit diverse origins, with distinct lineages fulfilling specific roles in the processes of repair and remodeling (46). The overall repair mechanism is contingent upon the precise regulation of anti-inflammatory signals (47) and the timely modulation of macrophage activation states (48). Using a murine model as an illustrative example, it has been observed that during the inflammatory resolution phase (approximately days 3 to 5), the concurrent administration of mRNA encoding interleukin-10 (IL-10) alongside either KLF4 or PPARγ facilitates the transition of macrophages from the pro-inflammatory M1 phenotype to the anti-inflammatory M2 phenotype. IL-10 is a cytokine known for its potent anti-inflammatory properties and its role in mitigating left ventricular remodeling through STAT3 activation (49). This temporal specificity has been elegantly validated by recent spatiotemporal tracking studies, which demonstrate that intravenously delivered mRNA-LNPs predominantly localize within myeloid immune cells during the initial three days following MI. Subsequently, these nanoparticles naturally alter their tropism towards cardiac fibroblasts and endothelial cells in the later stages of post-infarction (50). These findings underscore the critical importance of administering myeloid-targeted mRNAs precisely within this early temporal window.

The function of neutrophils presents a paradoxical nature, as they facilitate early tissue repair by directing macrophages towards a reparative phenotype (51) while concurrently contributing to inflammation through the release of neutrophil extracellular traps (NETs) (52, 53). Clinical data suggest that an elevated intracoronary NET burden is frequently correlated with larger infarct sizes and a more challenging resolution of inflammation (54). To mitigate this issue, the administration of DNase I mRNA during the acute neutrophil phase (days 0–2) enables the degradation of NETs within this critical period (55, 56). Both macrophage polarization and NET clearance strategies leverage the transient expression of mRNA to synchronize with the specific demands of each immune phase.

In the context of cardiovascular mRNA research, the metabolic dimensions of macrophage polarization remain insufficiently explored. M1 and M2 macrophages cannot be solely characterized by surface markers or cytokine profiles, as they engage in distinctly different metabolic pathways (57). M1 macrophages predominantly utilize aerobic glycolysis, commonly referred to as the Warburg effect, and display a disruption in the tricarboxylic acid (TCA) cycle. This disruption results in the accumulation of succinate, which subsequently stabilizes hypoxia-inducible factor 1-alpha (HIF-1α) and promotes the transcription of interleukin-1 beta (IL-1β) (58). Itaconic acid, another metabolite derived from the TCA cycle and produced by immune-responsive gene 1 (IRG1/ACOD1), demonstrates anti-inflammatory properties. Nonetheless, in the context of inflammation post-myocardial infarction, the predominance of glycolysis in M1 macrophages is likely to supersede this regulatory feedback mechanism (59).

Conversely, M2 macrophages depend on mitochondrial fatty acid oxidation (FAO) and maintain an intact oxidative phosphorylation (OXPHOS) chain. The rate-limiting step of FAO is modulated by carnitine palmitoyltransferase 1a (CPT1a), which facilitates the transport of long-chain fatty acids into the mitochondrial matrix (60). Building upon this mechanism, a promising therapeutic strategy involves the utilization of CD11b-targeted LNPs to deliver mRNA encoding CPT1a to cardiac macrophages. This approach facilitates a metabolic shift from glycolysis to FAO, thereby promoting M2 macrophage polarization at the metabolic level rather than through transcriptional modulation. This method is intended to complement existing cytokine-based strategies, such as those involving IL-10 mRNA, rather than merely replicating their mechanisms of action.

Previous research has demonstrated that the pharmacological activation of AMP-activated protein kinase (AMPK) can induce similar metabolic shifts *in vitro* (61). The delivery of mRNA encoding a constitutively active AMPK α-subunit can transiently replicate this effect. Compared to small-molecule AMPK activators, such as metformin or AICAR, mRNA offers the advantage of cell-specific targeting: while systemic AMPK activation impacts the entire organism, mRNA delivered through targeted LNPs can confine metabolic reprogramming to cardiac macrophages. This specificity is crucial, as systemic metabolic interventions may lead to adverse effects such as lactic acidosis and hypoglycemia, whereas the localized expression of mRNA can effectively mitigate these potential side effects.

Moreover, the integration of metabolic reprogramming (via CPT1a or AMPK mRNA) with transcriptional reprogramming (via IL-10 or KLF4 mRNA) within a dual-action LNP presents a promising approach for developing a synergistic ‘metabolic-transcriptional switch.’ This approach may offer superior efficacy compared to either strategy employed independently. It is imperative that future research prioritizes the validation of this combined strategy in a mouse model of myocardial infarction.

### 3.2 Lymphoid targets: transient CAR-T cells, regulatory T cells, and regulatory B cells

Current lymphocyte-based strategies for cardiac immunomodulation are centered around two well-established technical methodologies. The first strategy utilizes CD5-targeted mRNA-LNP to induce transient anti-fibrotic chimeric antigen receptor T (CAR-T) cells *in vivo*, aimed at the eradication of FAP-positive myofibroblasts (62). The second strategy involves the prospective *in situ* engineering of regulatory Tregs.

Tissue-resident Tregs are uniquely positioned to mitigate post-myocardial infarction inflammation and facilitate improved cardiac remodeling (63–65). While systemic immunotherapies utilizing Tregs have demonstrated promising outcomes in various diseases (66, 67), and engineered cytokine formulations such as IL-2 have been employed to augment the functionality of these cells (68), the concurrent delivery of Foxp3 and CXCR4 (the receptor for the cardioprotective factor SDF-1) via mRNA (69) presents a potential method for the transient expansion of these regulatory cells directly at the site of infarction (70, 71). Both methodologies leverage the inherently self-limiting nature of mRNA expression. Although the CAR-T strategy has been validated through *in vivo* experiments (62, 72), the Treg strategy necessitates further validation within cardiac models, despite its already robust mechanistic foundation.

The third lymphocyte population of interest comprises regulatory B cells (Bregs), which are known to secrete IL-10, IL-35, and TGF-β. These cells play a crucial role in modulating immune responses by suppressing the activity of inflammatory T cells and macrophages, either through direct cell-cell interactions or via cytokine release (73). In the realm of cardiac research, studies have demonstrated that the depletion of B cells using anti-CD20 antibodies exacerbates cardiac remodeling following myocardial infarction in murine models. Conversely, the adoptive transfer of IL-10-secreting B cells has been shown to enhance cardiac function. More recently, Cerqueira has reported a significant observation: the number of Bregs decreases sharply during the first week post-myocardial infarction, resulting in a gap in immune regulation. This period coincides precisely with the transition of the heart from the inflammatory phase to the repair phase (74).

The mRNA-LNP technology presents a promising avenue for addressing this gap. One potential strategy involves utilizing CD19-targeted LNPs to deliver IL-10 mRNA, thereby augmenting the IL-10 secretion capacity of existing B cells and effectively transforming conventional B cells into Breg-like effector cells. An alternative approach entails delivering mRNA encoding the transcription factor Blimp-1 (PRDM1), which directs B cells to differentiate into an IL-10-secreting phenotype (75). Due to the transient nature of mRNA, this reprogramming of B cells does not result in permanent impairment of humoral immunity, a concern associated with chronic B-cell regulation in autoimmune therapies.

Additionally, two other types of innate lymphoid cells merit brief consideration. γδ T cells infiltrate the infarct zone within hours following a myocardial infarction and produce IL-17A. The effects of this cytokine are distinctly dose- and time-dependent: early production of IL-17A facilitates neutrophil recruitment but becomes detrimental when excessive; subsequently, IL-17A supports fibroblast activation and wound closure (76). The second group of innate lymphoid cells (ILC2s) facilitates eosinophil recruitment and M2 macrophage polarization through the secretion of IL-5 and IL-13, both of which promote reparative functions (77).

However, the targeted delivery of mRNA to γδ T cells and ILC2s is currently impeded by technical challenges, primarily due to the absence of specific surface markers for LNP targeting. Nonetheless, with advancements in single-cell surface proteomics leading to the identification of novel targetable antigens, these cell populations are progressively being recognized as a promising area for future research.

### 3.3 The cardiomyocyte as a paracrine immunomodulatory hub

An innovative strategy in mRNA-LNP therapy involves transforming surviving cardiomyocytes in the ischemic border zone into immunoregulatory entities through paracrine reprogramming. Instead of directly targeting immune cells, this approach exploits the natural liver and heart tropism of certain LNP formulations to deliver mRNA to cardiomyocytes. This enables the cardiomyocytes to express secreted immunoregulatory proteins, which subsequently modulate the local immune microenvironment (78).

The most clinically advanced example of this approach is AZD8601, a modified mRNA encoding vascular endothelial growth factor A (VEGF-A_165_). In preclinical cardiac models, this therapeutic agent has exhibited dual functionalities in promoting angiogenesis and modulating immune responses (79, 80). Research conducted by Zangi et al. revealed that intracardiac administration of VEGF-A mRNA in a murine model of myocardial infarction facilitates neovascularization, reduces infarct size, and enhances survival rates (81). Beyond its traditional angiogenic roles, VEGF-A significantly influences the immune microenvironment by promoting monocyte chemotaxis, enhancing M2 macrophage polarization, and inhibiting dendritic cell maturation through VEGFR1-dependent signaling pathways (82). The combined angiogenic and immunomodulatory properties of VEGF-A underscore the multi-target potential of single-mRNA therapy in the treatment of post-myocardial infarction.

Building on this strategy, we propose the use of cardiomyocyte-mediated expression of soluble decoy receptor mRNA to neutralize pro-inflammatory cytokines within the infarcted region. In particular, the expression of soluble TNF receptor (sTNFR) or IL-1 receptor antagonist (IL-1Ra) by myocardial cells located in the border zone may establish a localized anti-inflammatory barrier. This barrier serves to protect the surviving myocardium from inflammatory damage while circumventing the systemic immunosuppression typically associated with systemic biologics (83). Utilizing cardiomyocytes for the expression of these proteins offers a distinct advantage over directly targeting immune cells, as cardiomyocytes are more amenable to LNP delivery, especially when formulations exhibit cardiac tropism. This approach facilitates higher concentrations of immunomodulatory proteins within the cardiac stroma (84).

## 4 The delivery conundrum: targeting immune vs. cardiac cells

The therapeutic efficacy of mRNA-LNP technology in cardiac immunomodulation is intrinsically linked to the precision of delivery. Unlike systemic delivery strategies employed in cancer therapy, which can target the liver or lymphoid tissues, cardiac therapy necessitates achieving two specific delivery goals: firstly, the precise targeting of specific immune cell subsets within the myocardium or those recruited to the myocardium; and secondly, the direct delivery to myocardial cells within the infarct zone and its adjacent regions. The requirement for dual-targeting of both immune cells and myocardial cells introduces distinct technical challenges and has spurred ongoing innovation in LNP engineering.

### 4.1 Redirecting LNP tropism

Conventional ionizable LNPs display a pronounced affinity for the liver after intravenous administration. This affinity is largely attributed to the binding of apolipoprotein E (ApoE) to the LNP surface, which facilitates uptake by hepatocytes through the low-density lipoprotein receptor (LDLR) (85, 86). While such affinity is beneficial for liver-targeted therapies, it presents significant challenges for biodistribution in cardiac immunotherapy. Quantitative analyses indicate that following systemic injection, the myocardium receives only a small fraction of the administered dose, typically less than one-tenth of that accumulated in the liver (87). As a result, achieving therapeutically significant mRNA expression in the heart via intravenous delivery often requires high doses of LNPs, thereby increasing the risk of systemic immunotoxicity and hepatotoxicity.

The Selective Organ Targeting (SORT) platform, developed by the Siegwart team, illustrates that the systematic incorporation of auxiliary lipid components into LNP formulations can effectively redirect tissue affinity from the liver to the spleen, lungs, or other organs (88). Specifically, the inclusion of permanently cationic lipids, such as DOTAP, enhances tropism towards the spleen and its resident immune cell populations, whereas the incorporation of anionic lipids facilitates drug delivery to the lungs. This precisely modulated organ-targeting strategy provides valuable insights for cardiac immunotherapy: targeting the spleen enables the transfection of monocyte precursors prior to their delivery to the infarcted heart, while lung-targeting formulations can capture immune cells circulating within the pulmonary vasculature (88).

Antibody-conjugated LNPs offer a more precise targeting strategy. The CD5-targeting LNP employed by Rurik et al. for *in vivo* CAR-T cell generation exemplifies this approach, achieving selective transduction of T cells via the conjugation of an anti-CD5 antibody (62).Building on this model, coupling LNPs with antibodies targeting macrophage-specific surface markers, such as CD11b, F4/80, CD68, or the macrophage mannose receptor CD206 in mice, facilitates the selective delivery of immunomodulatory mRNA to cardiac macrophages (89). This strategy effectively differentiates cardiac macrophages from those in other tissues. Specificity can be further enhanced by combining antibody targeting with localized (intramyocardial) delivery.

For direct cardiac delivery, intramyocardial injection during cardiac catheterization or open-heart surgery represents the most direct method, entirely circumventing mRNA retention due to the hepatic first-pass effect. The clinical program for AZD8601 (VEGF-A mRNA) demonstrated the clinical feasibility of direct cardiac mRNA delivery through epicardial injection during coronary artery bypass grafting (CABG) (90). For patients who are not candidates for surgery, emerging catheter-assisted endomyocardial injection techniques, such as the NOGA-guided MyoStar catheter system, present a minimally invasive strategy for the delivery of intramyocardial mRNA-LNPs (91). Nonetheless, despite the advantage of direct local injection, the overall delivery efficiency remains a significant translational challenge. Preclinical models indicate that the persistent mechanical contraction of the heart and rapid venous wash-out contribute to a considerable portion of the injected mRNA-LNP dose escaping into the systemic circulation, subsequently accumulating in the liver and spleen (92). Additionally, within the myocardium, LNPs primarily transfect interstitial fibroblasts, epicardial cells, and recruited immune cells, while the efficiency of *in vivo* cardiomyocyte transfection remains notably low (93). Addressing these biodistribution challenges to enhance on-target delivery efficiency, without resorting to highly invasive delivery methods, remains a critical barrier to routine clinical translation (**Table 2**).

**Table 2:** LNP targeting strategies for cardiac immunotherapy.

Strategy	Mechanism	Target cell(s)	Advantages	Limitations
Conventional IV LNP	ApoE, LDLR	Hepatocytes	Well-characterized	Liver sequestration (85, 86, 93)
SORT LNP (cationic)	DOTAP, spleen	Splenic immune cells	Pre-deploys to monocyte pool	Off-target splenic effects (88, 94)
CD5-targeted LNP	Anti-CD5 Ab	T lymphocytes	Proven *in vivo* CAR-T	Ab manufacturing complexity (62)
CD11b-targeted LNP	Anti-CD11b Ab	Cardiac macrophages	Direct myeloid targeting	Tissue specificity (89, 95)
Intramyocardial	Direct local delivery	Cardiomyocytes + local	Bypasses liver	Invasive (surgery/catheter) (90, 91, 96)
EV-LNP hybrids	EV membrane fusion	Cardiac cells, macrophages	Biomimetic, low immunogenicity	Scalability challenges (97, 98)

### 4.2 The immunogenicity paradox of LNPs in the infarcted heart

In the domain of cardiac mRNA therapy, a significant yet frequently underappreciated challenge is the immunogenicity of the LNP vector itself, particularly within the pro-inflammatory milieu of infarcted myocardium. While mRNA payloads can be rendered immunologically inert through nucleoside modification and purification methodologies, the lipid constituents of LNPs, specifically polyethylene glycol (PEG) lipids and ionizable lipids, possess the potential to elicit immune responses by activating the complement system and innate immune cells (99, 100).

Research conducted by Ndeupen et al. demonstrates that even in the absence of an mRNA payload, the intradermal or intranasal administration of LNPs provokes a pronounced inflammatory reaction, characterized by substantial neutrophil infiltration, activation of multiple inflammatory pathways, and the secretion of pro-inflammatory cytokines and chemokines (101). This intrinsic immunogenicity is predominantly attributed to the ionizable lipid component, which, upon endosomal uptake, activates the NLRP3 inflammasome by compromising lysosomal integrity; concurrently, the PEG-lipid component induces complement activation through alternative pathways (102, 103). Recent ex vivo studies have confirmed that standard vaccine-grade LNPs significantly increase complement activation products, such as C5a and sC5b-9, in human sera (104). Furthermore, mechanistic advancements in 2024–2025 have identified that specific amine headgroups in ionizable lipids can directly interact with and activate TLR4, leading to a substantial release of pro-inflammatory cytokines (105). Additionally, while endosomal rupture is necessary for mRNA escape, it inherently activates innate immune sensors. However, newly engineered LNPs, designed to minimize endosomal damage sensing, have effectively reduced this undesirable inflammatory signaling without compromising translation efficiency (106).

In this context, the intrinsic immunogenicity of LNPs presents a significant challenge in the post-myocardial infarction environment. The infarcted myocardium already demonstrates maximal complement activation through DAMP-mediated classical pathways, lectin pathways, neutrophil infiltration, and NLRP3 inflammasome activation (31, 107). The introduction of immunostimulatory LNPs into an already highly inflamed environment is likely to exacerbate tissue damage through a ‘second-hit’ mechanism, akin to the inflammation amplification patterns observed in sepsis and acute respiratory distress syndrome (108). Specifically, LNP-mediated complement activation leads to the production of anaphylatoxins C3a and C5a, which subsequently intensify neutrophil degranulation and reactive oxygen species (ROS) production. Concurrently, NLRP3 activation facilitates the release of IL-1β and IL-18, perpetuating the inflammatory cascade (109).

To mitigate this issue, several strategies are being investigated to reduce the immunogenicity of LNPs in cardiac applications. One approach involves the development of biodegradable, ionizable lipids, such as the ester-containing lipids utilized in Moderna’s SM-102 and Acuitas’s ALC-0315, which are designed to be rapidly metabolized and degraded following mRNA delivery. This approach aims to shorten the duration of immune cell activation and decrease the risk of inflammation (110). Second, LNP formulations that exclude PEG or incorporate PEG alternatives are currently under investigation. The widespread presence of PEG in everyday consumer products has led to the production of anti-PEG antibodies in up to 40% of the general population. This immune response accelerates the clearance of PEGylated nanoparticles from the bloodstream upon repeated administration, a phenomenon known as the accelerated blood clearance (ABC) phenomenon, and may also provoke complement-activated pseudoallergic reactions (CARPA) (111, 112). In contrast, alternative stealth polymers, such as poly-L-arginine, poly(2-oxazoline), and amphiphilic lipids, demonstrate pharmacokinetic profiles comparable to that of PEG but do not elicit anti-polymer antibody responses (113).

Third, the utilization of biomimetic delivery systems that mimic endogenous biological carriers presents a more direct approach to addressing this immunogenicity challenge. In recent years, extracellular vesicles (EVs) have gained prominence as significant biological models in the domain of drug delivery (114). EVs derived from cardiac progenitor cells or mesenchymal stromal cells have been shown to significantly enhance the microenvironment post-myocardial infarction by promoting angiogenesis and exerting anti-inflammatory effects (115). Consequently, these EVs inherently possess immunocompatible carrier properties (116, 117). Despite the technical challenges that remain in EV-based mRNA delivery, particularly concerning drug-loading efficiency and large-scale production, a hybrid system that integrates the mRNA packaging advantages of synthetic LNPs with the biocompatibility of EV membranes, referred to as a fusion LNP-EV hybrid, emerges as a promising solution (97).

In the assessment of delivery vehicles, a critical consideration involves the trade-off between LNPs and biodegradable polymeric nanoparticles (PNPs), such as poly(lactic-co-glycolic acid) (PLGA) or poly(beta-amino ester) (PBAE). Although PNPs generally demonstrate superior biocompatibility and biodegradability, resulting in significantly reduced systemic and cardiac toxicity compared to the highly immunogenic ionizable lipids, their *in vivo* transfection efficiency and capability for endosomal escape have historically been inferior to those of optimized LNPs (118). Recent advancements in lipid-polymer hybrid nanoparticles are designed to address existing challenges; however, LNPs continue to be the clinical benchmark for RNA delivery efficiency. This is despite their higher baseline immunogenicity, which necessitates careful management, particularly in the context of the infarcted heart (119).

### 4.3 Next generation payloads: self-amplifying RNA for extended expression

In the therapeutic balance between expression duration and safety, standard mRNA facilitates the production of therapeutic proteins within 24 to 72 hours. Although this transient ‘pulse’ is well-suited for acute intervention windows, such as early NET degradation, it fails to address the therapeutic needs of chronic remodeling processes that require sustained modulation over several weeks. In contrast, AAV vectors provide lifelong expression but pose irreversible risks. Self-amplifying RNA (saRNA) presents an optimal intermediate solution (120). By offering an intermediate expression duration, typically 3 to 4 weeks from a single injection, while maintaining the non-integrating and redosable characteristics of RNA, saRNA aligns well with the subacute structural remodeling phase of the infarcted heart. This approach mitigates the permanent immunosuppressive risks associated with viral vectors (121). Recent preclinical investigations have illustrated that intramuscular administration of saRNA encoding atrial natriuretic peptide (ANP) offers enhanced and prolonged cardioprotective effects compared to traditional mRNA, without eliciting systemic toxicity (21).

The saRNA is engineered utilizing an alphavirus replicon framework, typically sourced from the Venezuelan equine encephalitis virus, and encodes both the target therapeutic protein and a series of non-structural proteins (nsP1–nsP4) that serve as RNA-dependent RNA polymerases (122). Upon entry into the cytoplasm, saRNA is capable of self-replication, generating numerous copies independent of genomic integration. This mechanism facilitates sustained high-level protein expression with an initial dose significantly lower, by a factor of 10 to 100, than that required for conventional mRNA (120) In the context of cardiac applications, this reduction in dosage translates to a decreased requirement for LNPs per injection, thereby potentially minimizing LNP-associated immunogenic responses.

This challenge is particularly pronounced at the level of innate immune system activation. The double-stranded RNA intermediates generated during saRNA replication activate RIG-I, MDA5, and PKR, which are immune sensors that nucleoside-modified mRNAs aim to evade. In myocardial tissue that is already inflamed following a myocardial infarction, this additional type I interferon activation signal exacerbates the problem. Several strategies are currently under investigation to address this issue. Firstly, incorporating m1Ψ modifications into the saRNA backbone can diminish sensor recognition, although this may compromise replicon activity. Secondly, co-delivering innate immune-inhibiting proteins alongside saRNA within a lipid nanoparticle can locally suppress the interferon response while avoiding systemic immunosuppression (123). Thirdly, utilizing a trans-amplifying RNA system separates the replicase and therapeutic target into two independent RNA molecules, thereby reducing the accumulation of double-stranded RNA intermediates (124).

### 4.4 Comparative perspectives on alternative emerging delivery technologies

While LNPs currently predominate in mRNA therapeutics, a comparative analysis of emerging alternative delivery platforms, such as EVs, PNPs, and hydrogels, reveals distinct advantages and limitations for cardiac immunomodulation.

As endogenous biological carriers, EVs, particularly those derived from mesenchymal stem cells, offer superior biocompatibility, significantly lower immunogenicity, and inherent tissue-homing capabilities across biological barriers when compared to synthetic LNPs (125). Nonetheless, the clinical translation of EV-based mRNA therapies is significantly hindered by challenges such as low exogenous RNA loading efficiency, high batch-to-batch variability, and the complexities associated with standardized, large-scale Good Manufacturing Practice (GMP) production, which are areas where LNPs currently maintain a definitive industrial advantage (126).

Biodegradable polymers, such as PLGA and PBAEs, present a safer alternative to the highly inflammatory ionizable lipids utilized in traditional LNPs. PNPs can substantially mitigate systemic and cardiac toxicity due to their physiological degradation pathways (127). Nevertheless, the *in vivo* endosomal escape and mRNA transfection efficiencies of these systems have historically been inferior to those of optimized LNPs, prompting the development of hybrid lipid-polymer systems to effectively balance efficacy and safety (128).

To address the significant liver sequestration and systemic off-target immunotoxicity associated with intravenous LNP administration, the delivery of mRNA-loaded nanoparticles via injectable shear-thinning or thermosensitive hydrogels presents an attractive localized strategy. These hydrogels establish a sustained-release depot directly at the site of the infarcted myocardium or within the pericardial cavity, thereby limiting systemic exposure, reducing LNP-related systemic immunogenicity, and facilitating prolonged localized immune modulation over several weeks (129, 130).

As discussed in the introduction, CRISPR-Cas9 genome editing provides an unparalleled method for the permanent ablation of stable pathogenic targets, such as PCSK9 mutations or genetic cardiomyopathies (131). Nevertheless, the immune response following myocardial infarction, which swiftly transitions from acute inflammation to resolution, necessitates transient and highly reversible modulation. The permanent genetic deletion of inflammatory pathways using CRISPR technology currently lacks the necessary temporal flexibility and poses long-term safety concerns, such as impaired infection surveillance. Consequently, transient mRNA systems emerge as a more appropriate tool for phase-specific, reversible cardiac immunological engineering (10).

## 5 Bridging preclinical success to clinical reality

The transition of mRNA-LNP therapeutics from preclinical cardiac models to clinical application has advanced through two primary pathways: AZD8601 (VEGF-A mRNA) developed by AstraZeneca/Moderna and mRNA-0184 (relaxin mRNA) developed by Moderna. Although these programs target distinct pathological mechanisms, collectively they illuminate the clinical challenges and immunological considerations associated with cardiac mRNA therapy (**Table 3**).

**Table 3:** Summary of commercial and clinical mRNA/LNP therapeutic strategies and safety concerns.

Program/strategy	Sponsor	Target/payload	Delivery route	Indication	Phase	Key safety concerns/limitations	References
AZD8601	AstraZeneca/Moderna	VEGF-A_165_	Epicardial injection	Ischemic HD	Phase II	Invasive delivery requirement; local myocardial edema. Systemic tolerance is good but requires open-chest or catheter delivery.	(79, 80, 96)
mRNA-0184	Moderna	Relaxin-2 (LNP)	Intravenous	HFrEF	Phase I	Systemic exposure risks; liver sequestration; ABC effect upon repeat dosing; potential immune activation from LNP.	(132)
VERVE-101/YOLT-101	Verve Therapeutics/Others	PCSK9 CRISPR base editing	IV LNP (hepatic)	FH/Atherosclerosis	Phase Ib	Permanent off-target editing risks; pre-existing Cas immunity; transient LNP-driven transaminitis.	(10, 133–135)
mRNA-6231	Moderna	Relaxin analog	Intravenous LNP	Heart failure	Phase I	Similar to mRNA-0184: balancing systemic LNP immunogenicity with required repeated infusions	(136, 137)

### 5.1 AZD8601: VEGF-A mRNA for ischemic heart disease

AZD8601 consists of a modified mRNA encoding VEGF-A165, formulated in a citrate-buffered saline solution, allowing for naked mRNA uptake via direct intramyocardial injection, as it is not encapsulated in LNPs. A Phase IIa clinical trial (NCT03370887) involving patients undergoing CABG demonstrated the safety and tolerability of epicardial injection, with secondary endpoints indicating improvements in ventricular wall motion and myocardial perfusion in the treated area (96, 138).

A significant immunological observation from this trial was that intramyocardial injection did not elicit a substantial systemic inflammatory response, suggesting that local mRNA delivery may circumvent the systemic immunogenicity issues typically associated with intravenous administration of LNP-encapsulated mRNA (96, 136). The selection of VEGF-A as a therapeutic target underscores the dual mechanisms of angiogenesis and immune regulation previously discussed. The outcomes of this trial are anticipated to yield crucial insights into the immunological impacts of mRNA therapy on the human heart.

### 5.2 mRNA-0184 and repeated dosing challenges

Moderna’s mRNA-0184 encodes the hormone relaxin-2, with its recombinant form known as serelaxin, encapsulated in a LNP for intravenous administration in patients with heart failure. Serelaxin-2 exerts vasodilatory, antifibrotic, and anti-inflammatory effects through the RXFP1 receptor. Although recombinant serelaxin demonstrated certain hemodynamic benefits in the RELAX-AHF trial (139), it did not achieve a reduction in 180-day mortality in the larger RELAX-AHF-2 trial (140). In contrast, mRNA technology presents potential advantages, such as enabling sustained protein production (thereby circumventing the rapid clearance associated with recombinant serelaxin) and facilitating repeated dosing (132).

The Phase I clinical trial of mRNA-0184 (NCT04916431) serves as a pivotal validation study for systemic LNP-mediated mRNA delivery in patients with heart failure. These patients frequently exhibit chronic low-grade inflammation, elevated baseline complement activity, and compromised liver function, all of which may affect the biodistribution of LNPs (141). Critical immunological endpoints include the assessment of complement activation levels (C3a, C5a, sC5b-9), biomarkers linked to cytokine release syndrome (CRS) (such as IL-6, IFN-γ, and ferritin), and the development of anti-polyethylene glycol (PEG) antibodies. The results of this study will contribute to defining the therapeutic window for intravenous mRNA-LNP therapy in individuals with cardiovascular disease.

In contrast to vaccination, which generally necessitates only one or two doses, cardiac mRNA therapies may require repeated administrations, particularly in the management of chronic heart failure or for multiple antifibrotic interventions. The immunological challenges associated with repeated dosing differ significantly from those encountered with a single dose. Specifically, anti-PEG antibodies generated after initial exposure to lipid nanoparticles (LNPs) induce an accelerated blood clearance (ABC) phenomenon. This results in subsequent doses being rapidly processed and eliminated by splenic and hepatic macrophages, thereby substantially diminishing therapeutic efficacy (111). Studies utilizing animal models have demonstrated that administering a second dose within a two-week interval can lead to a reduction in LNP-mediated gene expression by more than 90% due to the ABC effect (142).

**Figure 3:**
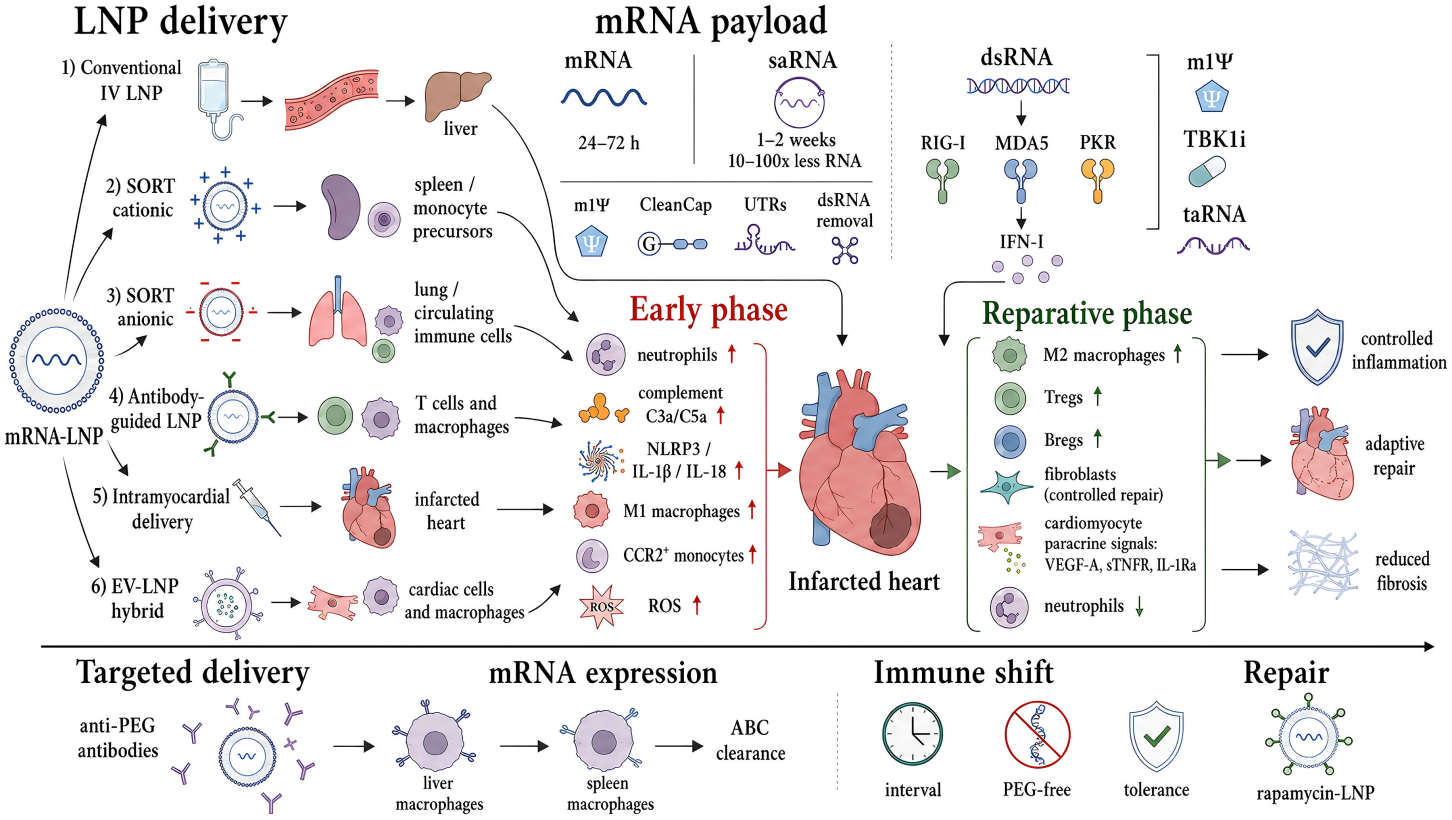
Targeted LNP delivery routes, RNA payload kinetics, and strategies to overcome ABC clearance in post-MI immunotherapy.

To address the ABC effect, several strategies are currently under investigation: (i) Extending the dosing interval to exploit the relatively short half-life of IgM anti-PEG antibodies, thereby administering the subsequent dose only after a natural decline in antibody titers (143); (ii) Transitioning to PEG-free formulations, as previously discussed; (iii) Employing tolerogenic nanoparticles for pretreatment to induce antigen-specific immune tolerance to PEG (144); (iv) Incorporating rapamycin into LNP formulations to induce localized immunosuppression and prevent immune responses against the carrier (145). The optimal strategy may vary depending on the clinical setting: acute MI intervention may require only 1–2 doses (thereby minimizing the impact of the ABC effect), whereas chronic heart failure treatment may necessitate PEG-free or tolerance-inducing regimens (**Figure 3**).

### 5.3 Biomarker guided precision dosing: timing mRNA intervention

The optimal strategy may differ according to the clinical context: for instance, intervention in acute myocardial infarction might require only one to two doses, thereby minimizing the impact of the ABC effect. In contrast, treatment for chronic heart failure may necessitate PEG-free or tolerance-inducing regimens (**Figure 3**).

The essence of spatiotemporal immune reprogramming lies in administering the appropriate mRNA at the precise moment. However, how can clinicians ascertain this optimal timing? At present, there are no validated point-of-care tests available to determine whether a patient’s immune response following a myocardial infarction has progressed from the inflammatory phase to the resolution phase. The development of such biomarkers is essential before mRNA immunotherapy can be integrated into routine clinical practice (5).

Circulating biomarkers present the most convenient method for detection. Continuous monitoring of the ratio of CCR2-positive to CCR2-negative monocytes through flow cytometry can effectively track the transition from the inflammatory phase to the reparative phase (3). While the dynamic changes in high-sensitivity C-reactive protein (CRP) and IL-6 are relatively rudimentary indicators, they offer practical utility: the decline in IL-6 levels following their peak on the second day generally signifies the commencement of inflammation resolution. Galectin-3 and soluble ST2 (sST2) have been employed in the prognostic evaluation of heart failure and can also function as real-time markers of fibrotic activity, thereby informing dose adjustments for anti-fibrotic CAR-T therapies (146). In the realm of imaging, T2-weighted cardiac magnetic resonance imaging (MRI) can quantify myocardial edema, indicative of active inflammation, while T1-weighted imaging based on extracellular volume (ECV) fractions can monitor the progression of fibrosis (147). Fluorodeoxyglucose positron emission tomography (FDG-PET) is capable of detecting metabolically active macrophages in infarct regions; however, the associated radiation exposure constrains its feasibility for continuous monitoring (148).

Liquid biopsy technology is progressively advancing toward clinical implementation. Circulating free DNA (cfDNA) with myocardial-specific methylation signatures can reflect ongoing cell death processes and assist in identifying patients whose inflammatory phase persists beyond the typical duration (149). Exosomal microRNA testing introduces an additional dimension: the ratio of circulating miR-155, indicative of M1 macrophage activity, to miR-223, associated with inflammation resolution, has been suggested as a potential marker for disease staging. However, this requires prospective validation within mRNA therapy trials (150). Looking forward, we foresee the development of point-of-care devices that integrate multiple biomarkers, including monocyte phenotypes, cfDNA methylation status, and miRNA panel testing, to produce a comprehensive ‘immunostaging score’. This score would aid in determining the optimal mRNA combination therapy regimen for each patient at every clinical visit.

Beyond determining the optimal timing, patient stratification remains a significant clinical challenge, as demographic variables significantly influence host immune responses to LNP-mRNA therapies. Elderly myocardial infarction patients often exhibit baseline ‘inflammaging’ and immunosenescence, characterized by delayed phagocytosis and inherently elevated pro-inflammatory cytokines, such as IL-6 and TNF-α. As a result, these patients may demonstrate diminished therapeutic responses or increased susceptibility to adverse inflammatory cascades triggered by immune-activating mRNAs (151, 152). In contrast, clinical data from COVID-19 mRNA vaccines indicate that younger male patients are at a significantly higher risk for LNP-associated myocarditis, suggesting that differences in immune reactivity may be driven by sex hormones (153, 154). Additionally, metabolic comorbidities such as diabetes and chronic kidney disease intensify baseline inflammation, necessitating personalized dosing strategies and distinct target selections, such as prioritizing anti-fibrotic pathways over anti-inflammatory ones in cases of advanced diabetes (155). Addressing these stratification challenges will likely require the integration of multi-omics approaches and the use of circulating non-coding RNA panels to accurately predict patient-specific responses prior to mRNA administration (156, 157).

### 5.4 Critical limitations: scalability, stability, and regulatory hurdles

Although the clinical prospects for cardiac mRNA therapies are promising, several translational challenges must be critically addressed. While large-scale vaccine production has significantly reduced standard mRNA-LNP manufacturing costs to approximately $1 to $4 per dose (137, 158), the development of tailored or personalized mRNA therapies, such as specific anti-fibrotic multicomponent formulations, encounters substantial financial challenges. The formulation of individualized, small-batch interventions necessitates costly IVT raw materials, including capping analogs, modified nucleosides, and enzymes, and does not benefit from economies of scale (159). To render personalized cardiac mRNA therapies economically feasible, a shift from centralized large-batch production to adaptable, continuous microfluidic assembly platforms is imperative (160).

Additionally, the stringent cold-chain requirements present a significant obstacle. Standard liquid LNP formulations necessitate storage at ultra-low temperatures (≤-20 °C) to maintain long-term integrity, as storage at standard refrigeration (4 °C) or room temperature results in rapid LNP aggregation, mRNA degradation, and a marked decline in *in vivo* transfection efficiency within months (161, 162). Advancements in next-generation lyophilization techniques, which employ rationally screened cryoprotectants such as sucrose or trehalose, have demonstrated the capability to preserve the structure and transfection efficacy of LNP formulations for extended periods at room temperature. This represents a significant milestone for the broader application of cardiac clinical therapies (163–165).

However, the regulatory framework for cardiac mRNA immunotherapy remains to be clearly defined. Current therapeutic approaches must navigate intricate Chemistry, Manufacturing, and Controls (CMC) requirements. Unlike single-molecule drugs with well-defined characteristics, LNP-mRNA complexes necessitate stringent regulation of Critical Quality Attributes (CQAs), including mRNA integrity, dsRNA impurities, and consistency in lipid composition across different batches (136, 166). Looking ahead, recent regulatory and analytical advancements highlight the importance of standardized frameworks initially developed for personalized neoantigen mRNA vaccines. Techniques such as rapid liquid chromatography for assessing poly(A) tail length and stringent limits on dsRNA impurities are now being adopted as foundational quality control measures for the development of emerging cardiovascular mRNA therapies (167).

## 6 Conclusion and future perspectives

The integration of mRNA therapy with cardiac immunology represents a pivotal advancement in treatment strategies for post-myocardial infarction ventricular remodeling, transitioning from conventional, broad-spectrum pharmacological immunosuppression to precise spatiotemporal immune reprogramming. This review delineates three fundamental innovations: the attainment of phase-matched immune regulation through the transient expression kinetics of mRNA; the dual application of effector cells and regulatory T cells via *in vivo* immune cell engineering; and the identification and resolution of the immunogenicity paradox associated with LNPs in inflamed cardiac tissue. Collectively, these innovations establish a novel therapeutic paradigm that fully exploits the unique advantages of the mRNA-LNP platform to effectively address the immunological complexities inherent in cardiac healing.

In the future, it is anticipated that cardiac mRNA therapies will progress towards multi-component ‘cocktail’ formulations that can simultaneously target multiple immune pathways. For instance, an mRNA cocktail encapsulating VEGF-A (angiogenic), IL-10 (anti-inflammatory), and Foxp3 (regulatory) could harmonize repair signals across the domains of angiogenesis, innate immunity, and adaptive immunity within a single treatment (168). Current research has demonstrated the technical feasibility of co-encapsulating multiple mRNAs within a single LNP; leveraging this advancement, the development of combination vaccines emerges as a logical subsequent step (169).

A critical factor that is frequently overlooked, yet pivotal in determining the efficacy of personalized mRNA therapies, is biological sex. Notably, there are significant differences in the immune responses of male and female patients following myocardial infarction: premenopausal women exhibit a more robust IFN-I immune response, higher regulatory T cell counts, and reduced neutrophil infiltration during the acute phase. While these characteristics may facilitate earlier healing, they also elevate the risk of microvascular inflammation and diffuse fibrosis, which can ultimately lead to heart failure with preserved ejection fraction (HFpEF) (170). Conversely, male patients tend to exhibit more pronounced neutrophil and M1 macrophage infiltration, a higher risk of ventricular rupture, and more severe acute myocardial remodeling (171). The estrogen receptor α signaling pathway in macrophages promotes M2 polarization, an effect that diminishes after menopause (172).

These differences have direct implications for the design of mRNA combination therapies. Male patients may derive the greatest benefit from early anti-NET therapy (DNase I mRNA) and aggressive treatment strategies aimed at promoting M1-to-M2 conversion (IL-10 or CPT1a mRNA). Female patients, particularly postmenopausal women in whom estrogen-driven M2 polarization has ceased, may require less IFN-I inhibition and instead benefit more from targeted anti-fibrotic interventions. Incorporating gender-stratified dosing regimens based on the previously mentioned biomarker combinations is essential for the design of future clinical trials concerning cardiac mRNA immunotherapies.

The integration of spatial transcriptomics with mRNA therapy presents substantial potential for advancing precision cardiac immunotherapy. Analyzing single-cell and spatial transcriptomic profiles of human myocardial infarction regions reveals significant heterogeneity at both cellular and molecular levels within the infarct core, border zone, and distal myocardium, with each region displaying a distinct composition and activation status of immune cells (173, 174). These findings inform the rational design of mRNA therapies; for instance, IL-10 mRNA can be specifically delivered to target anatomical regions to enhance the expression of anti-inflammatory mediators in the penumbra. Concurrently, the infarct core, which is predominantly occupied by neutrophils and pro-inflammatory macrophages during the acute phase, could benefit from the administration of DNase I mRNA to degrade NETs (175).

The advent of *in situ* spatial transcriptomics platforms, such as Visium, MERFISH, and Slide-seq, facilitates the real-time mapping of the spatial landscape of immune responses following myocardial infarction at a single-cell resolution. These spatial maps hold potential as companion diagnostic tools, aiding in the determination of timing, dosage, and target selection for mRNA immunotherapies (176). We propose a future framework termed ‘spatio-temporal immunomapping-guided therapy’, which leverages spatial transcriptomic analyses of endomyocardial biopsy samples or employs non-invasive molecular imaging techniques to develop personalized mRNA combination therapy regimens and to ascertain the optimal timing of administration for each patient.

To achieve this vision, it is imperative to address several key challenges. Firstly, the translation of cardiac-targeted LNP formulations from murine models to larger animal models and human applications necessitates systematic optimization. This is due to interspecies differences in immune cell receptor expression, complement pathway activity, and cardiac anatomy, which can markedly affect delivery efficiency and immunological outcomes (177). Secondly, overcoming obstacles related to manufacturing scalability, stringent cold-chain requirements, and the evolving regulatory landscape for multi-component mRNA cocktails remains a considerable challenge (136, 178). Thirdly, as these therapies progress into advanced stages of clinical development, rigorous long-term safety monitoring becomes crucial. This is particularly important in relation to the generation of anti-PEG antibodies from repeated dosing, potential autoimmune complications, impaired tumor surveillance, and LNP-induced acute inflammation (151, 179).

In conclusion, mRNA-LNP therapies present a distinctive opportunity to modify the cardiac immune microenvironment following myocardial infarction with precise spatiotemporal control, thereby converting the body’s immune system into a therapeutic instrument for cardiac repair. Advancing from the current stage of preclinical research to clinical implementation necessitates interdisciplinary collaboration among fields such as immunology, cardiology, bioengineering, and pharmaceutical science. Nonetheless, the transformative potential of this approach to alter the detrimental post-MI immune environment from a contributor to heart failure into a facilitator of cardiac regeneration underscores its intrinsic research significance.

## Glossary

MI: Myocardial infarction
mRNA: Messenger RNA
LNPs: Lipid nanoparticles
CAR-T: Chimeric Antigen Receptor T cells
Tregs: Regulatory T cells
SORT: Selective Organ Targeting
IL-1β: Interleukin-1β
AAV: Adeno-associated virus
m1Ψ: N1-methylpseudouridine
COVID-19: Coronavirus disease 2019
saRNA: Self-amplifying RNA
Ab: Antibody
DAMPs: Damage-associated molecular patterns
HMGB1: High mobility group box 1
ATP: Adenosine triphosphate
PRRs: Pattern recognition receptors
Ψ: Pseudouridine
TLR (TLR3: TLR7, TLR8), Toll-like receptors
RIG-I: Retinoic acid-inducible gene I
MDA5: Melanoma Differentiation-Associated protein 5
IFN-I: Type I interferon
IFN-α: Interferon-alpha
IFN-β: Interferon-beta
PKR: Protein kinase R
OAS: 2’-5’-oligoadenylate synthetase
eIF2α: Eukaryotic initiation factor 2 alpha
dsRNA: Double-stranded RNA
IVT: *In vitro* transcription
HPLC: High-performance liquid chromatography
NLRP3: NLR family pyrin domain containing 3
UTRs: Untranslated regions
CCR2: C-C chemokine receptor type 2
cGAS-STING: Cyclic GMP-AMP synthase-stimulator of interferon genes
H3K4me3: H3K4 trimethylation
H3K27ac: H3K27 acetylation
TNF-α: Tumor necrosis factor alpha
IL-6: Interleukin-6
HSPCs: Hematopoietic stem and progenitor cells
LDL: Low-density lipoprotein
TET2: Ten-eleven translocation methylcytosine dioxygenase 2
KDM5A: Lysine demethylase 5A
IL-10: Interleukin-10
KLF4: Krüppel-like factor 4
PPARγ: Peroxisome proliferator-activated receptor gamma
NETs: Neutrophil extracellular traps
DNase I: Deoxyribonuclease I
TCA: Tricarboxylic acid
HIF-1α: Hypoxia-inducible factor 1-alpha
IRG1/ACOD1: Immunoresponsive gene 1/Aconitate decarboxylase 1
FAO: Fatty acid oxidation
OXPHOS: Oxidative phosphorylation
CPT1a: Carnitine palmitoyltransferase 1a
AMPK: AMP-activated protein kinase
AICAR: 5-Aminoimidazole-4-carboxamide ribonucleotide
FAP: Fibroblast activation protein
CXCR4: C-X-C chemokine receptor type 4
SDF-1: Stromal cell-derived factor 1
Foxp3: Forkhead box P3
Bregs: Regulatory B cells
IL-35: Interleukin-35
TGF-β: Transforming growth factor beta
Blimp-1 (PRDM1): B lymphocyte-induced maturation protein-1/PR/SET domain 1
IL-17A: Interleukin-17A
ILC2s: Group 2 innate lymphoid cells
IL-5: Interleukin-5
IL-13: Interleukin-13
VEGF-A: Vascular endothelial growth factor A
VEGFR1: Vascular endothelial growth factor receptor 1
sTNFR: Soluble TNF receptor
IL-1Ra: IL-1 receptor antagonist
ApoE: Apolipoprotein E
LDLR: Low-density lipoprotein receptor
DOTAP: 1,2-dioleoyl-3-trimethylammonium-propane
CD (CD5: CD11b, CD19, CD20, CD68, CD206), Cluster of differentiation
CABG: Coronary artery bypass grafting
PEG: Polyethylene glycol
ROS: Reactive oxygen species
IL-18: Interleukin-18
ABC: Accelerated blood clearance
CARPA: Complement-activated pseudoallergic reactions
EVs: Extracellular vesicles
nsP (nsP1-nsP4): Non-structural proteins
HD: Heart disease
HFrEF: Heart failure with reduced ejection fraction
PCSK9: Proprotein convertase subtilisin/kexin type 9
FH: Familial hypercholesterolemia
RXFP1: Relaxin family peptide receptor 1
CRS: Cytokine release syndrome
IFN-γ: Interferon-gamma
CRP: C-reactive protein
sST2: Soluble ST2
MRI: Magnetic resonance imaging
ECV: Extracellular volume
FDG-PET: Fluorodeoxyglucose-positron emission tomography
cfDNA: Circulating free DNA
miRNA/miR: MicroRNA
HFpEF: Heart failure with preserved ejection fraction
M1: Classically activated macrophages
M2: Anti-inflammatory macrophages
PNPs: Polymeric nanoparticles
ANP: Atrial natriuretic peptide
GMP: Good Manufacturing Practice
PLGA: Poly(lactic-co-glycolic acid)
PBAE: Poly(beta-amino ester)
STAT3: Signal transducer and activator of transcription 3
CaMKIIδ: Calcium/calmodulin-dependent protein kinase II delta
CMC: Chemistry, Manufacturing, and Controls
CQAs: Critical Quality Attributes
IV: Intravenous
IgM: Immunoglobulin M
CRISPR: Clustered regularly interspaced short palindromic repeats
Cas9: CRISPR-associated protein 9
C3a/C5a: Complement component 3a/5a
sC5b-9: Soluble terminal complement complex
